# Supplementary material for: Economic burden of brain metastases in non-small cell lung cancer patients in South Korea: A retrospective cohort study using nationwide claims data
Source: PLoS One. 2022 Sep 20;17(9):e0274876. doi: 10.1371/journal.pone.0274876 (PMC9488774; doi:10.1371/journal.pone.0274876)
Supplement: Attachment — Submitted filename: PONE-D-21-30124_Review.docx [file pone.0274876.s001.docx]

This manuscript identifies an economic burden of brain metastases in non-small cell lung cancer patients in South Korea using nationwide claims data. This study is timely considering the trend in increasing prevalence of non-small cell lung cancer patients in nationwide. So, this study will be good addition to the literature for the future reader. However, the manuscript will need to develop and require more revision based on my comments.

The authors must provide the rationale for selection of covariates based on either theory-driven or literature including categorization of your independent variables.

This manuscript used the South Korea’s claims data. However, I didn’t see any epidemiological information in South Korea in the introduction. Please add one paragraph of the information about South Korea.

The authors provided USD monetary unit in the paper. Did you convert monetary unit from South Korea to USD in the paper? If so, please indicate this information in the manuscript.

The policy implication of this study can be further addressed; the discussion section should be further developed specially limitation section (i.e., generalizability issue).

For the quality of written English: Acceptable level, but overall, careful proof reading by an expert English writer would assist identifying and revision the article

The methods section is very clear and provides rationale and evidence for each of the data sources used. The analysis is clearly described.

Otherwise, well-written manuscript.

Level of interest: An article of importance in its field!!
