# Supplementary material for: Economic burden of brain metastases in non-small cell lung cancer patients in South Korea: A retrospective cohort study using nationwide claims data
Source: PLoS One. 2022 Sep 20;17(9):e0274876. doi: 10.1371/journal.pone.0274876 (PMC9488774; doi:10.1371/journal.pone.0274876)
Supplement: Attachment — Submitted filename: Response to Reviewers.docx [file pone.0274876.s002.docx]

We appreciate the thoughtful comments provided by the reviewers, and we believe that the manuscript has been strengthened in response to their comments. In the following pages, we provide a point-by-point response (blue) to their comments (black). We described changes to the reference lists following the responses.

**Reviewer #1**: This manuscript identifies an economic burden of brain metastases in non-small cell lung cancer patients in South Korea using nationwide claims data. This study is timely considering the trend in increasing prevalence of non-small cell lung cancer patients in nationwide. So, this study will be good addition to the literature for the future reader. However, the manuscript will need to develop and require more revision based on my comments.

1. The authors must provide the rationale for selection of covariates based on either theory-driven or literature including categorization of your independent variables.
Thanks to your suggestions, we added references which studied impact of brain metastases on economic burden in NSCLC. We referred to those studies when selecting key covariates in our analysis. Added sentence and references are in Method section line 133 as follows:

*To compare the demographic and clinical characteristics of the patients with and without BM before and after PS matching, we estimated the distribution of patients’ sex, age, non-cancer-related CCI, death, other cancers, and follow-up days for each group. We included these covariates based on previous retrospective studies that examined the economic impact of BM in NSCLC using claims data [22, 23].*

2. This manuscript used the South Korea’s claims data. However, I didn’t see any epidemiological information in South Korea in the introduction. Please add one paragraph of the information about South Korea.
Thanks to your comment, we searched for more studies on epidemiological results of brain metastases in NSCLC. As a result, we could add more references of brain metastases incidence and a reference of South Korea which showed more than 10% of advanced staged NSCLC patients developed brain metastases. Added sentences are in the introduction section line 53 as follows:

*Several studies have reported that brain metastases (BM) occur in > 10% of NSCLC patients during disease progression [8-11]. In South Korea, 10.9% of advanced-stage NSCLC patients develop BM within two years of initial diagnosis [12].*

3. The authors provided USD monetary unit in the paper. Did you convert monetary unit from South Korea to USD in the paper? If so, please indicate this information in the manuscript.

Thanks to your comment, we could clarify the monetary unit we used and how we converted it. We edited the sentence which was describing the conversion of monetary unit with more specification in the Method section line 160 as follows:

*All costs were converted from South Korean won to 2019 US dollars using the exchange rate (1166 South Korean won = 1 US dollar) obtained from the Korean Statistical Information Service.*

4. The policy implication of this study can be further addressed; the discussion section should be further developed specially limitation section (i.e., generalizability issue).

Thanks to your comment, we could develop the discussion section. We compared our study with previous studies in terms of cost differences due to brain metastases in NSCLC and more costs incurred in inpatient settings than in outpatient settings in the discussion section line 256 and 268, respectively. Moreover, we added interpretation of higher costs in younger patients in the discussion section line 292.

Added sentences are as follows:

line 256

*Previous studies have also shown the economic burden of BM. Fernandes et al., in a study of patients with NSCLC using epidermal growth factor receptor tyrosine kinase inhibitors, reported that the healthcare cost after BM diagnosis increased significantly compared with that before BM diagnosis (US$ 9670 vs. US$ 17 581) [17]. Similarly, the monthly cost per patient was US$ 5983 before the diagnosis of BM but increased markedly to US$ 22 645 after BM in a US study based on patients treated with crizotinib [19]. The cost difference observed in these studies was greater than that observed in our study. Since healthcare costs increase as disease severity worsens [26, 27], before-and-after comparisons used in previous studies might have included costs due to disease progression in the costs after diagnosis. Since we ruled out the possibility of cost increase due to disease progression by comparing patients with and without BM, our result would represent the economic burden of BM itself.*

line 268

*In our study, PPPM costs in patients with BM comprised more inpatient costs than outpatient costs. This is consistent with previous studies that showed more PPPM costs in inpatient settings than in outpatient settings in patients with BM [22, 23]. Moreover, as medications and radiation therapy have been suggested as the main cost drivers in patients with BM [22], drug costs comprised the greatest part of the total costs, followed by radiology/radiotherapy in our study. These greatest cost components were incurred in both inpatient and outpatient settings, with drug costs incurred mostly in outpatient settings and radiology/radiotherapy costs incurred mostly in inpatient settings.*

*Longer PPPM length of hospital stay (3.0 vs. 1.2 days) and more frequent outpatient visits (1.7 vs. 1.4 times) in anaplastic lymphoma kinase (ALK) inhibitor-treated NSCLC patients with BM compared to those without BM were also observed in a previous study [22]. Compared to the study, our results showed a higher PPPM length of hospital stay and outpatient visits in patients with and without BM. This could be caused by the clinical circumstances of South Korea, in which relatively more healthcare resources are used than in other countries under fee-for-service systems [24].*

Line 292

*Consistent with a previous study that indicated that younger lung cancer patients aged less than 65 years had higher medical costs compared to patients aged ≥ 65 years [28], our GLM analysis showed that younger patients incur higher healthcare costs. It could be inferred that higher costs were incurred because younger age has been reported to be associated with more tolerable and aggressive cancer treatments [29, 30]. Likewise, higher costs of targeted therapy, ICI, and radiology/radiotherapy were incurred in younger patients in our study.*

In the limitation section, we added descriptions that differences between countries should be considered when generalizing our results into global level and there is lack of information about the characteristics of the tumor in the data that we used. Added sentences are in the discussion section line 317 and 326 as follows:

Line 317 and 326:

*This study has several limitations. First, the HIRA claims data are recorded as reimbursed services only. Therefore, drugs and treatments not covered by insurance were not identified in this study.* *Second, there was a lack of information on the characteristics of tumors in the HIRA data. No information on the disease progression or clinical stage of cancer was available. In addition, the ICD-10 codes, the diagnostic codes from the HIRA database, do not distinguish between NSCLC and small cell lung cancer. To improve the accuracy in defining patients with NSCLC, we consulted clinicians on the relevant diagnostic and procedure codes for NSCLC and used drugs and treatments exclusively for patients with NSCLC [20]. Information on the diagnoses, procedures, and drugs used might have been inaccurate due to misclassification; however, a previous validation study that compared HIRA data and actual medical records of hospitalized patients reported a concordance of 82% [32]. Lastly, although our study results highly represent the economic burden of NSCLC and BM patients in South Korea using HIRA claims data, differences between countries should be considered when generalizing our results to the global level.*

5. For the quality of written English: Acceptable level, but overall, careful proof reading by an expert English writer would assist identifying and revision the article
Thank you for your thoughtful suggestion. We requested English editing and revised English expressions throughout the manuscript. Please find the attached certificate of English editing.

6. The methods section is very clear and provides rationale and evidence for each of the data sources used. The analysis is clearly described.
We sincerely appreciate your positive feedback.

Otherwise, well-written manuscript.
Level of interest: An article of importance in its field!!

**Reviewer #2**: The study on the economic burden of brain metastases in NSCLC in South Korea is very interesting. It’s a well-designed study, however, I have some minor comments that the authors may want to address.

Minor comments
• Abstract (page 2, line 22): Adding 1-2 sentence(s) about the background/rationale of this study will give your audience better perspective about its importance.

Thanks to the constructive comments, we added two sentences to make the audience more interested in our study. We added the sentences in the abstract section line 21 as follows:

*Brain metastases (BM) are common in patients with non-small cell lung cancer (NSCLC). However, the pure economic burden of BM is unknown.*

• Abstract (page 2, line 34): The authors used the term “hospitalization days” throughout the manuscript. This language does not match with today’s literature. More appropriate language would be “length of hospital stay.”

Thanks to your comment, we changed all the terms into “length of hospital stay” throughout the manuscript.

• Abstract (page 2, line 38): The term “principal components” makes the description confusing because the results didn’t come from principal components analysis or unsupervised learning. I would suggest using other terms for GLM or what the authors used.

We changed the expression of the sentence thanks to your suggestion in abstract section line 37 as follows:

*The costs of drugs, radiology/radiotherapy, and admission comprised the majority of PPPM costs and were higher in patients with BM.*

• Introduction (page 3, lines 47-48): please add a reference.

Thank you for your comment. The sentences in line 44-47 are from the same reference, therefore, the reference is placed in the introduction section line 47 as follows:

*Lung cancer is common cancer that has the second highest annual incidence rate among cancers. An estimated 2.2 million new patients were diagnosed with lung cancer in 2020 worldwide. Lung cancer is also the leading cause of cancer-related deaths, resulting in 1.8 million deaths per year worldwide [1].*

• Introduction (page 3, lines 54-56): If possible, it would be better to have worldwide data and Korean data rather than the U.S and Korea. This would make it consistent with previous information, as well as show the current situation in Korea.

Thanks to your comment, we added a reference which showed incidence of brain metastases (BM) among NSCLC patients in South Korea. Moreover, we added two more references to show that studies consistently showed more than 10% of BM occurrence in NSCLC. Added references and sentences are the introduction section line 53 as follows:

*Several studies have reported that brain metastases (BM) occur in > 10% of NSCLC patients during disease progression [8-11].* *In South Korea, 10.9% of advanced-stage NSCLC patients develop BM within two years of initial diagnosis [12].*

• Introduction (page 3, lines 59-60): Talking about the QOL seems out of the scope of this paper.

Thanks to your suggestion, we deleted the sentence.

• Introduction (page 3, lines 47-60): If possible, it would be better to have information regarding Korea rather than the U.S.

Thank you for your comment. We added epidemiological information about the incidence of brain metastasis in NSCLC in South Korea in the introduction section line 54 as follows:

*In South Korea, 10.9% of advanced-stage NSCLC patients develop BM within two years of initial diagnosis [12].*

• Introduction (page 4, line 67): For reference 14, please specify which county’s results.

We specified that the results were from US in introduction section line 63 as follows:

*A US study observed a large increase in healthcare costs in patients with epidermal growth factor receptor (EGFR)-mutated NSCLC and BM from 2012 to 2015, compared with patients with metastases at other sites [17].*

• Introduction (page 4, line 67): “using US claims data” – The authors used “United States” previously. Please be consistent.

Thanks to your comment, we consistently used “US” throughout the manuscript.

• Introduction (page 4, lines 69-72): “Studies have also ….” Please add references.

Thanks to your comment, we added references to the sentence in the introduction section line 67 as follows:

*Studies have shown the impact of BM on the increase in economic burden either by comparing patients before and after their diagnosis of BM [18, 19] or by comparing patients with BM and patients with metastases at other sites [17].*

• Introduction (page 4, lines 69-72): “However, previous studies have also ….” Please add references.
We added the references to the sentence in introduction section line 68 as follows:

*However, these previous studies [17-19] have used designs that include not only the cost of BM but also the increasing cost of disease progression, thereby leaving the pure economic burden of BM compared with the economic cost in the absence of BM not studied.*

• Methods: The words “code C34” and “code C793” were repeated many times. Once a term has been defined, it does not need to be defined again. Deleting duplicate descriptions will aid readability and decrease unnecessary word use.

Thanks to your suggestion, after first defining, we deleted the words “code C34” and “code C793” when they were not necessary.

• Methods (page 8, line 162): “...radiology/radiotherapy, and other costs.” Please specify what types of costs were included in the other category.

Thanks to your suggestion, we edited the sentence by listing the types of costs according to the sequence listed in Table2. We classified costs into 7 types (drugs, dispensing, admission, radiology/radiotherapy, tests, procedures, consultations) and the rest of costs were classified as others. The edited sentence is in line 154 as follows:

*Healthcare costs included the costs of drugs, dispensing, admission, radiology/radiotherapy, tests, procedures, consultations, and others.*

• Discussion (page 19, lines 336-344): Additional limitations to consider include generalizability beyond the study sample, and the lack of information about the characteristics of the tumor.

Thanks to your comment, we developed our discussion section in terms of lack of information of cancer characteristics (e.g., disease progression, clinical stage) and generalizability issue when interpreting our results into global level. We added the sentences in the discussion section line 317 and 326 as follows:

line 317

*Second, there was a lack of information on the characteristics of tumors in the HIRA data. No information on the disease progression or clinical stage of cancer was available.*

line 326

*Lastly, although our study results highly represent the economic burden of NSCLC and BM patients in South Korea using HIRA claims data, differences between countries should be considered when generalizing our results to the global level.*

**Reviewer #3**:

1. What is the unit of 3.64 vs. 3.40 in the abstract (page 2, page 37)

We intended to express the mean number of visits. Thanks to your comment, we clarified the unit in the abstract section line 36 and result section line 224 as follows:

*Abstract section:* *patients with BM had more outpatient visits (50.61 vs. 46.43 times for two years, p<.0001; 3.64 vs. 3.40 times PPPM costs, p<.0001).*

*Results section:* *Further, patients with BM had more outpatient visits than patients without BM (50.61 vs. 46.43 times, p<.0001 for two years; 3.64 vs. 3.40 times, p<.0001 for PPPM).*

2. In the abstract, the author said “It is important to prevent BM in patients with NSCLC to reduce the economic burden on society.” But this study only estimated the medical cost from the healthcare system perspective, not societal perspective. Is this interpretation appropriate?

Thanks for the meaningful comment. As in the comments, this study was analyzed from the health care system perspective, so the interpretation of 'on society' may be inappropriate. Therefore, the phrase 'on society' was deleted in the abstract and conclusion section.

3. Minor: page3, line 61, please use the abbreviations NSCLC and BM, because the authors already spelled out and abbreviated those words.

Thanks to your comment, we edited the sentence using the abbreviations in the introduction section line 57 as follows:

*NSCLC poses a heavy economic burden on society, especially in terms of healthcare resources [16]. BM constitutes this burden because of the additional treatment required for brain lesions.*

4. Page 5, line 93: What is the meaning of “From the index date, patients were followed up for up to 2 years or until the date were removed”

We intended to explain patients are followed up from the index date to whichever comes first among 2 years after the index date or the end of data (February 29, 2020). Thanks to your comment, we clarified the expression in method section line 89 as follows:

*We followed the patients from the index date until the last claim for lung cancer or metastatic cancers within two years after the index date.*

5. Immuno-cancer drugs such as pembrolizumab and atezolizumab are also used for NSCLC, but why is there no selection criteria?

We appreciate your detailed comments. We could confirm that nivolumab, pembrolizumab, and atezolizumab (immune checkpoint inhibitors) are covered for non-small cell lung cancer (NSCLC) patients who “failed previous platinum-based chemotherapy” in South Korea. Since we intended to include “newly diagnosed stage IIIB-IV NSCLC patients”, we did not include the use of ICI for inclusion criteria for “new” patients.

6. According to table3, the younger the age group, the higher the medical cost tends to be. Add an interpretation of this to the discussion.

Thanks to your suggestion, we added discussion about more costs incurred in younger patients in discussion section line 292 as follows:

*Consistent with a previous study that indicated that younger lung cancer patients aged less than 65 years had higher medical costs compared to patients aged ≥ 65 years [28], our GLM analysis showed that younger patients incur higher healthcare costs. It could be inferred that higher costs were incurred because younger age has been reported to be associated with more tolerable and aggressive cancer treatments [29, 30]. Likewise, higher costs of targeted therapy, ICI, and radiology/radiotherapy were incurred in younger patients in our study.*

7. In the introduction section, the examples of treatment options used for brain metastases (BM) in NSCLC should be explained to support increased economic burden from additional treatment needed for BM.

We appreciate your suggestion. We added examples of treatment options for brain metastases in introduction section line 59 as follows:

*Stereotactic radiosurgery, whole-brain radiation therapy, operational resection, and mutation-targeting therapies (e.g., tyrosine kinase inhibitors and immune checkpoint inhibitors) have been suggested as treatments for BM in NSCLC [9].*

8. Please how you enabled presenting “the pure economic burden of BM compared with the absence of BM” unlike other previous studies in the Introduction section.

We clarified the expressions in introduction section and added a sentence in the introduction section line 71 that describes how our study enabled identifying the pure economic burden of BM compared with the absence of BM as follows:

*Studies have shown the impact of BM on the increase in economic burden either by comparing patients before and after their diagnosis of BM [18, 19] or by comparing patients with BM and patients with metastases at other sites [17]. However, these previous studies [17-19] have used designs that include not only the cost of BM but also the increasing cost of disease progression, thereby leaving the pure economic burden of BM compared with the economic cost in the absence of BM not studied. Comparing the healthcare costs of patients with and without BM would show the pure economic impact of BM with increased costs due to disease progression in both groups.*

9. When referring to previous studies in the second paragraph of the Discussion section, please relate the studies with your results. The explanation about French study seems unrelated with your results.

Thank you for your comments, we revised the second paragraph in the discussion section substantially. We compared the results of previous studies and our study. Greater cost differences were reported in those previous studies and we emphasized the difference is the result of different methodology used. Before-and-after comparison that was used in previous studies could have included costs due to disease progression. Therefore, we believe our results are more applicable when describing the pure economic impact of BM in NSCLC. We edited second paragraph of discussion starting from line 256 as follows:

*Previous studies have also shown the economic burden of BM. Fernandes et al., in a study of patients with NSCLC using epidermal growth factor receptor tyrosine kinase inhibitors, reported that the healthcare cost after BM diagnosis increased significantly compared with that before BM diagnosis (US$ 9670 vs. US$ 17 581) [17]. Similarly, the monthly cost per patient was US$ 5983 before the diagnosis of BM but increased markedly to US$ 22 645 after BM in a US study based on patients treated with crizotinib [19]. The cost difference observed in these studies was greater than that observed in our study. Since healthcare costs increase as disease severity worsens [26, 27], before-and-after comparisons used in previous studies might have included costs due to disease progression in the costs after diagnosis. Since we ruled out the possibility of cost increase due to disease progression by comparing patients with and without BM, our result would represent the economic burden of BM itself.*

10. Please compare the results of your study and previous studies in terms of main items that comprised larger healthcare costs in BM patients in the third paragraph of the Discussion section.

Thanks to your suggestion, we edited the third paragraph of discussion section substantially. Our results showed results consistent with previous studies which showed that more costs were incurred in inpatients setting and the greatest components of cost were drug and radiology/radiotherapy. We edited the paragraph of discussion starting from line 268 as follows:

*In our study, PPPM costs in patients with BM comprised more inpatient costs than outpatient costs. This is consistent with previous studies that showed more PPPM costs in inpatient settings than in outpatient settings in patients with BM [22, 23]. Moreover, as medications and radiation therapy have been suggested as the main cost drivers in patients with BM [22], drug costs comprised the greatest part of the total costs, followed by radiology/radiotherapy in our study. These greatest cost components were incurred in both inpatient and outpatient settings, with drug costs incurred mostly in outpatient settings and radiology/radiotherapy costs incurred mostly in inpatient settings.*

*Longer PPPM length of hospital stay (3.0 vs. 1.2 days) and more frequent outpatient visits (1.7 vs. 1.4 times) in anaplastic lymphoma kinase (ALK) inhibitor-treated NSCLC patients with BM compared to those without BM were also observed in a previous study [22]. Compared to the study, our results showed a higher PPPM length of hospital stay and outpatient visits in patients with and without BM. This could be caused by the clinical circumstances of South Korea, in which relatively more healthcare resources are used than in other countries under fee-for-service systems [24].*

**Changes in the reference list**

The following references have been added during the revision:

10.	Schouten LJ, Rutten J, Huveneers HA, Twijnstra A. Incidence of brain metastases in a cohort of patients with carcinoma of the breast, colon, kidney, and lung and melanoma. Cancer. 2002;94(10):2698-705. doi: 10.1002/cncr.10541. PubMed PMID: 12173339.

11.	Jens B. Sorensen, Heine H. Hansen, Mogens Hansen, Dombernowsky P. Brain Metastases in Adenocarcinoma of the Lung: Frequency, Risk Groups, and Prognosis. Journal of Clinical Oncology. 1988;6:1474-80.

12.	Kim M, Suh CH, Lee SM, Park JE, Kim HC, Kim SO, et al. Development of Brain Metastases in Patients With Non-Small Cell Lung Cancer and No Brain Metastases at Initial Staging Evaluation: Cumulative Incidence and Risk Factor Analysis. AJR Am J Roentgenol. 2021;217(5):1184-93. Epub 2021/05/27. doi: 10.2214/ajr.21.25787. PubMed PMID: 34037408.

28.	Banegas MP, Yabroff KR, O'Keeffe-Rosetti MC, Ritzwoller DP, Fishman PA, Salloum RG, et al. Medical Care Costs Associated With Cancer in Integrated Delivery Systems. J Natl Compr Canc Netw. 2018;16(4):402-10. doi: 10.6004/jnccn.2017.7065. PubMed PMID: 29632060.

29.	Walter J, Tufman A, Holle R, Schwarzkopf L. "Age matters"-German claims data indicate disparities in lung cancer care between elderly and young patients. PLoS One. 2019;14(6):e0217434. Epub 20190612. doi: 10.1371/journal.pone.0217434. PubMed PMID: 31188861; PubMed Central PMCID: PMCPMC6561547.

30.	Yoneyama R, Saji H, Kato Y, Kudo Y, Shimada Y, Kimura M, et al. Clinicopathological characteristics and treatment strategies for young lung cancer patients. Ann Transl Med. 2019;7(5):100. doi: 10.21037/atm.2019.01.69. PubMed PMID: 31019950; PubMed Central PMCID: PMCPMC6462655.
